# A Treatise on Massage

**Published:** 1903-05

**Authors:** 


A TREATISE ON MASSAGE. Its History, Mode of Application and Effects, Indications and Contraindications. By DOUGLAS GRAHAM, M. D., Boston. Third edition revised, enlarged and illustrated. Octavo, pp. 462. Philadelphia and London: J. B. Lippincott Company. 1902. [Price, $4.00.]

The first edition of this work was published in 1884, and was the first book on massage in point of time in the English language. Since then the progress made has been considerable and has called forth new editions of this able work. The range of usefulness of massage has been steadily increasing and has long since extended into every special and general branch of medicine, so that it is important for the physician who desires to keep pace with its development to be well informed in all its special lines. This volume not only describes the correct methods of applying massage, but deals with the specific treatment of various disorders and diseases of the whole human system. Thus each disease is taken up in order, and the indications and contraindications pointed out with the special technic most advisable in each case. In looking through the list of diseases in which massage is indicated, it is found beneficial to some extent in nearly every disease to which mankind is heir, external as well as internal.

In nervous diseases especially it has been found exceedingly useful, and in none more so than in neurasthenia. Graham gives special attention to the application of massage in this disease, the relative value as met with in either sex, and those cases where it does little if any good. In rheumatism, gout, hepatic and renal disorders, it indicates a line of treatment of great benefit to remedial procedures.

It is in all a valuable book for the physician to possess,—just at the present time when physical therapeutic methods are attracting so much attention. The illustrations are excellent; paper and binding are very satisfactory. W. C. K.

